# Effects of *Porphyromonas gingivalis* LipopolysaccharideTolerized Monocytes on Inflammatory Responses in Neutrophils

**DOI:** 10.1371/journal.pone.0161482

**Published:** 2016-08-18

**Authors:** Xiang-qing Zhu, Wei Lu, Yang Chen, Xiao-fan Cheng, Jia-ying Qiu, Yan Xu, Ying Sun

**Affiliations:** 1 Jiangsu Key Laboratory of Oral Diseases, Nanjing Medical University, Nanjing, China; 2 Department of Periodontology, Affiliated Hospital of Stomatology, Nanjing Medical University, Nanjing, China; 3 Department of Stomatology, Nanjing First Hospital, Nanjing, China; Universita degli Studi di Napoli Federico II, ITALY

## Abstract

Periodontitis is a chronic inflammatory disease induced by bacteria. Exposure of the host to periodontal pathogens and their virulence factors induces a state of hyporesponsiveness to subsequent stimulations, which is termed endotoxin tolerance. The role and mechanism of lipopolysaccharide (LPS)–tolerized monocytes in inflammatory responses in neutrophils are currently unclear. Here, conditioned supernatants were collected from THP-1 cells treated with or without repeated 1 μg/ml *Porphyromonas gingivalis* (*P*.*gingivalis*) LPS. The chemotactic response of freshly isolated neutrophils recruited by supernatants was determined by a transwell migration assay, which demonstrated a reduced migration of neutrophils stimulated with supernatants from tolerized THP-1 cells in comparison to non-tolerized THP-1 cells. In addition, there was a marked increase in reactive oxygen species (ROS) generation and a significant decrease in Caspase 3 activities in neutrophils treated with supernatants from THP-1 cells that were treated repeatedly with *P*.*gingivalis* LPS in comparison to single treatment. A cytokine antibody array was then used to assess cytokine expression patterns in THP-1 cells. In tolerized THP-1 cells, 43 cytokine (43/170) expression levels were decreased, including chemokine ligand 23 (CCL23) and IFN-γ, while 11 cytokine (11/170) expression levels were increased, such as death receptor 6 (DR6). Furthermore, there was decreased production of IFN-γ and epithelial neutrophil activating peptide-78 (ENA-78) in THP-1 cells after stimulation with repeated *P*. *gingivalis* LPS in comparison to single challenge, which was confirmed by ELISA. Therefore, *P*.*gingivalis* LPS- tolerized THP-1 cells were able to depress neutrophil chemotaxis and apoptosis, and contribute to respiratory burst, which might be related to the changes in cytokine expression patterns in THP-1 cells.

## Introduction

Periodontitis is a chronic infectious disease, which is characterized by the loss of supporting tissues. It is one of the two major oral diseases in humans and is difficult to treat [1]. Bacteria have been considered to be the initiating factors to trigger periodontitis and *Porphyromonas gingivalis* (*P*.*gingivalis*), a gram-negative anaerobic bacterium, has been implicated to be one of the most important periodontal pathogens [2]. The cell wall components of *P*.*gingivalis*, especially lipopolysaccharide (LPS), can activate host immune responses, including the production of pro-inflammatory cytokines, anti-inflammatory cytokines and chemokines, which might be important in periodontitis development [3].

Bacterial stimulation on periodontal tissues is persistent in periodontitis development and exposure of the host to periodontal pathogens in addition to the associated virulence factors could induce a state of hyporesponsiveness to subsequent stimulations, which is called endotoxin tolerance. Accumulating evidence suggests that endotoxin tolerance is a reprogramming of the immune system, which results in decreased levels of pro-inflammatory cytokines, including TNF-α and IL-1β, and increased production of anti-inflammatory cytokines, such as IL-10 [4, 5]. Inhibiting excessive release of inflammatory mediators could be utilized to restrict immune injury and maintain periodontal homeostasis. Therefore, this could act as a protective mechanism in the progression of periodontitis. On the other hand, inhibiting inflammatory mediator release could also compromise the host's ability to resist subsequent invading bacteria [6]. Up to now, the exact roles and mechanisms of endotoxin tolerance in periodontitis still remain obscure.

Monocytes/macrophages and neutrophils are two different types of innate immune cells that act against intruding bacteria. After periodontal pathogens successfully overcome epithelial barriers and invade soft tissues, signals from bacteria and resident macrophages in the infected area activate local endothelial cells and guide neutrophils across endothelial cell lining. These neutrophils then produce chemokines, which attract additional neutrophils, macrophages and T cells to trigger the innate immune response and subsequent acquired immune response [7, 8]. Therefore, crosstalk may exist between neutrophils and monocytes/macrophages. Monocytes/macrophages are important innate immune cells at infection sites in patients with chronic periodontitis, and these cells produce large amounts of pro-inflammatory cytokines, anti-inflammatory cytokines and chemokines, including TNF-α, IL-1ß, IL-8 and IL-10. Previous studies indicated that monocytes/macrophages were involved in endotoxin tolerance [9]. Decreased production of TNF-α, IL-1ß and IL-6, coupled to an increase in IL-10 secretion was observed in LPS-tolerized monocytes [5, 9]. However, the effects of tolerized monocytes on inflammatory responses in neutrophils have yet to be elucidated.

In this study, we investigated the roles of *P*.*gingivalis* LPS–tolerized monocytes, THP-1 cells, in neutrophil migration, apoptosis and respiratory burst. In addition, changes in cytokine expression profiles in tolerized THP-1 cells were explored to reveal possible mechanisms for the above-mentioned changes in neutrophils.

## Materials and Methods

### Reagents

*P*.*gingivalis* ATCC 33277 LPS was purchased from InvivoGen (CA, USA). *Escherichia coli* (*E*.*coli*) 0111:B4 LPS and dichlorofluorescein diacetate (DCFH-DA) were obtained from Sigma Aldrich (MI, USA). Fluorescein Active Caspase 3 Staining Kit was supplied by Biovision (CA, USA). Recombinant human IL-8 was obtained from Peprotech (NJ, USA) and ELISA kits were purchased from R&D (MN, USA). Human cytokine antibody arrays (G-Series 2000) were purchased from Ray Biotech. (GA, USA).

### Cell Culture

This study was approved by the Ethical Committee of Nanjing Medical University in accordance with institutional guidelines (Permit Number: 20130204) and written informed consents were obtained from all recruits.

THP-1 cells (catalogue number: TCHu 57) were purchased from Shanghai Institutes for Biological Sciences, Chinese Academy of Sciences (Shanghai, China) in Sep 2014. Neutrophils were freshly isolated from healthy volunteers’ blood by Polymorphprep (Axis-Shielld, Norway) as previously described [10]. This procedure yielded a neutrophil population that was close to 95% pure and more than 90% viable, as assessed by flow cytometry. Both of these two types of cells were suspended in RPMI-1640 (Gibco, USA) supplemented with 10% fetal calf serum (Hyclone, USA) at 37°C in a humidified 5% CO_2_ atmosphere.

### Endotoxin Tolerance Induction

THP-1 cells (5×10^5^ cells/ml) were cultured in 6-well plates and divided into 5 groups (n = 5 per group). Group 1 was incubated in medium alone. Group 2 and 4 were cultured in media for 24 h, washed with PBS, and stimulated with medium containing 1 μg/ml *P*.*gingivalis* LPS or 1 μg/ml *E*.*col*i LPS for 24 h, respectively. Group 3 and 5 were treated with medium containing 1 μg/ml *P*.*gingivalis* LPS or 1 μg/ml *E*.*coli* LPS for 24 h, washed, then resuspended in medium containing 1 μg/ml *P*.*gingivalis* LPS or 1 μg/ml *E*.*coli* LPS for an additional 24 h, respectively. Cell free supernatants from tolerized and non-tolerized THP-1 cells were collected by centrifugation and stored at -80°C for subsequent experiments.

### Chemotaxis Assay

Chemotaxis was evaluated using 24-transwell chamber of 3μm pores size for neutrophils (Millpore, USA). Conditioned medium from tolerized or non-tolerized THP-1 cells was used as a chemoattractant in the lower chamber and aliquots of neutrophils (1×10^6^ cells/well) were added in the upper chamber. Medium supplemented with 100 ng/ml IL-8 served as the positive control. Blank culture medium and medium containing 1 μg/ml *P*.*gingivalis* LPS or 1 μg/ml *E*.*coli* LPS served as negative controls. After incubation for 90 min at 37°C, the filters were removed, fixed with ethanol and then stained with crystal violet [11]. Neutrophils migrating through polycarbonate membrane to its lower face were counted in 5 views per membrane under a phase contrast microscope. The results were expressed as chemotactic index, which was the number of cells that migrated towards the sample divided by the number of cells that migrated towards blank culture medium.

### Neutrophil Oxidative Burst

Neutrophils (10^6^ cells/ml) were cultured in 6-well plates and challenged with supernatants from tolerized or non-tolerized THP-1 cells for 4 h. Medium containing 1 μg/ml *P*.*gingivalis* LPS or 1 μg/ml *E*.*coli* LPS served as positive controls, and blank culture medium served as a negative control. The cells were then collected and incubated with 5 μM DCFH-DA for 40 min at 37°C. Intracellular reactive oxygen species (ROS) was measured using the nonfluorescent probe, DCFH-DA, which could penetrate into the intracellular matrix of cells, where it was oxidized by ROS to fluorescent DCF [12]. The cells were analyzed using a FACSCalibur (BD Biosciences, USA) and fluorescence intensities were expressed as percentages relative to the values of the cells treated with blank culture medium, which were normalized to 100%.

### Apoptosis Assessment

Freshly isolated neutrophils were cultured at a density of 10^6^ cells/ml in 6-well plates and stimulated with supernatants from tolerized or non-tolerized THP-1 cells for 5 h. Medium containing 1 μg/ml *P*.*gingivalis* LPS or 1 μg/ml *E*.*coli* LPS served as positive controls, and blank culture medium served as a negative control. Then, neutrophils were collected, resuspended in 300 μl PBS with 1 μl Caspase 3 inhibitor, FITC-DEVD-FMK, and incubated for 0.5 h at 37°C. After this incubation, Caspase positive cells were washed and analyzed by flow cytometry using the FL-1 channel. The results were expressed as percentages relative to the values of the cells treated with blank culture medium, which were normalized to 100%.

### Microarrays for Cytokines

A total of 170 cytokines in the culture medium from *P*.*gingivalis* LPS- tolerized, non-tolerized and non-stimulated THP-1 cells (group 1, 2 and 3) were screened by microarrays. 1 μl of each sample was used for analysis. Detection was performed by Kangcheng Biotechnology Company (Shanghai, China) according to the manufacturer’s instructions.

### Cytokine Detection by ELISA

Levels of IFN-γ and epithelial neutrophil-activating peptide 78 (ENA-78) in the supernatants from THP-1 cells were determined by ELISA kit according to the manufacturer’s protocol.

### Statistical Analysis

Statistical analysis was performed using ANOVA and LSD test was used to compare differences between groups. Data are expressed as mean±SD. P values less than 0.05 were considered to be statistically significant.

## Results

### Decreased Chemotaxis of Neutrophils Stimulated with Supernatants from Tolerized-THP-1 Cells

In order to investigate the effects of conditioned medium from tolerized THP-1 cells on neutrophil migration, the number of neutrophils that migrated through a transwell membrane toward the lower chamber was counted.

**Fig 1:**
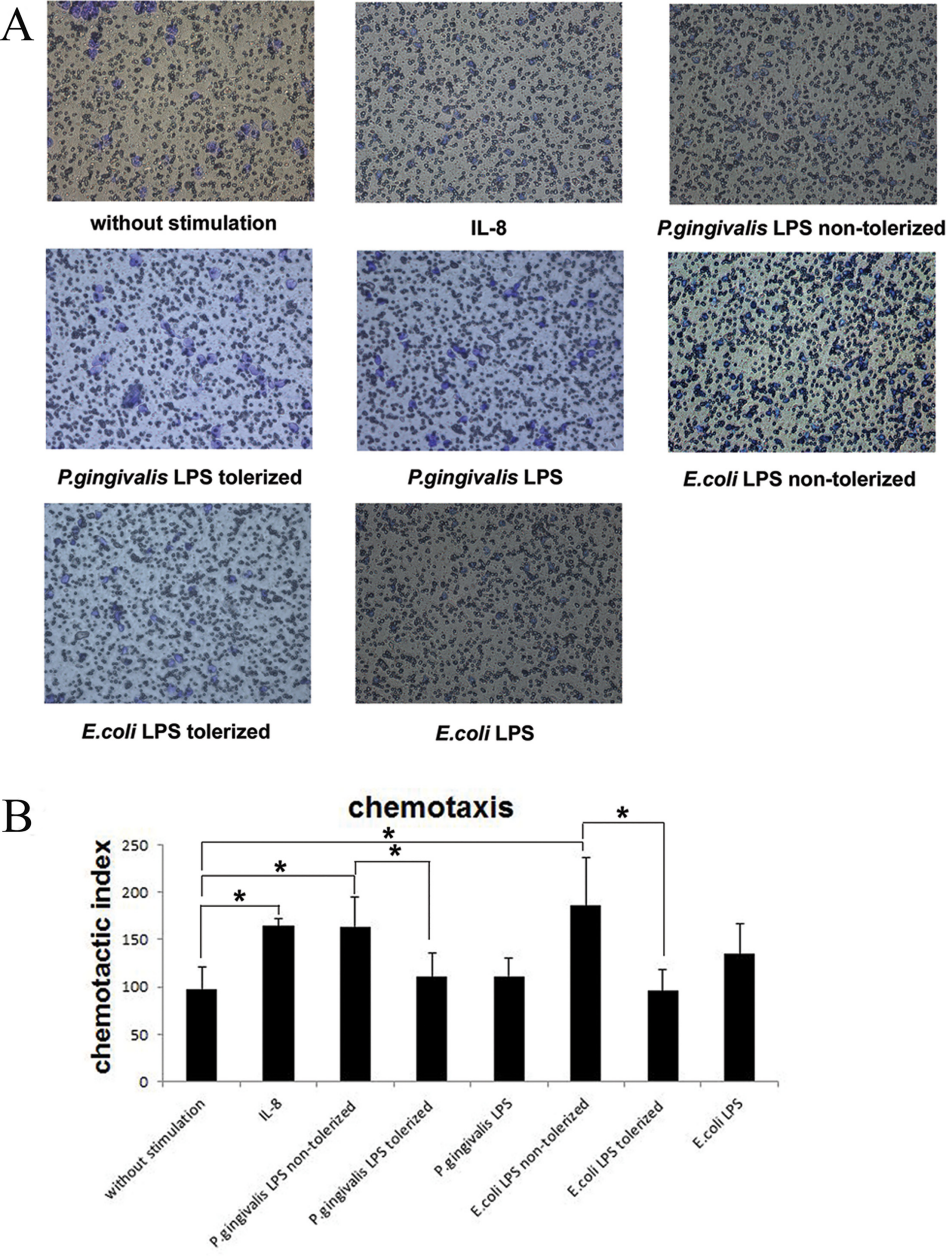
Effects of tolerized THP-1 cells on neutrophil migration. Neutrophils were resuspended in the upper chamber of a transwell plate and supernatants from 1 μg/ml *P*.*gingivalis* LPS or 1 μg/ml *E*.*coli* LPS tolerized/non-tolerized THP-1 cells were added to the lower chamber. *P*.*gingivalis* LPS, *E*.*coli* LPS and IL-8 were served as controls. Following incubation for 90 min, neutrophils that migrated through transwell membrane were counted in 5 fields under a microscope (×400). Data are expressed as mean±SD (n = 5 per group). *p<0.05. One representative result of five independent experiments is shown in (1A).

In comparison to neutrophils challenged with cultured medium from THP-1 cells that were not stimulated (group 1), there was an increase in neutrophils that migrated to the supernatants from THP-1 cells stimulated with 1 μg/ml *P*.*gingivalis* LPS (group 2) or 1 μg/ml *E*.*coli* LPS (group 4) only once (p<0.05), and this increase could also be observed in neutrophils challenged with 100 ng/ml IL-8 (p<0.05). However, following the stimulation of supernatants from *P*.*gingivalis* LPS- tolerized THP-1 cells (group 3), neutrophils that migrated toward the lower chamber was much less than those treated with conditioned culture medium from non-tolerized THP-1 cells (group 2) (p<0.05), and these same changes could also be observed in neutrophils stimulated with supernatants from *E*.*coli* LPS- tolerized (group 5) and non- tolerized THP-1 cells (group 4) (p<0.05) (Fig 1).

Importantly, there were no differences in neutrophils challenged with *P*.*gingivalis* LPS/*E*.*coli* LPS and the cultured medium from THP-1 cells without any stimulation (p>0.05), which implied that activating factors that led to neutrophil migration might be secreted by THP-1 cells but not *P*.*gingivalis* LPS or *E*.*coli* LPS (Fig 1).

### Increased ROS Production in Neutrophils Is Influenced by Tolerized THP-1 Cells

**Fig 2:**
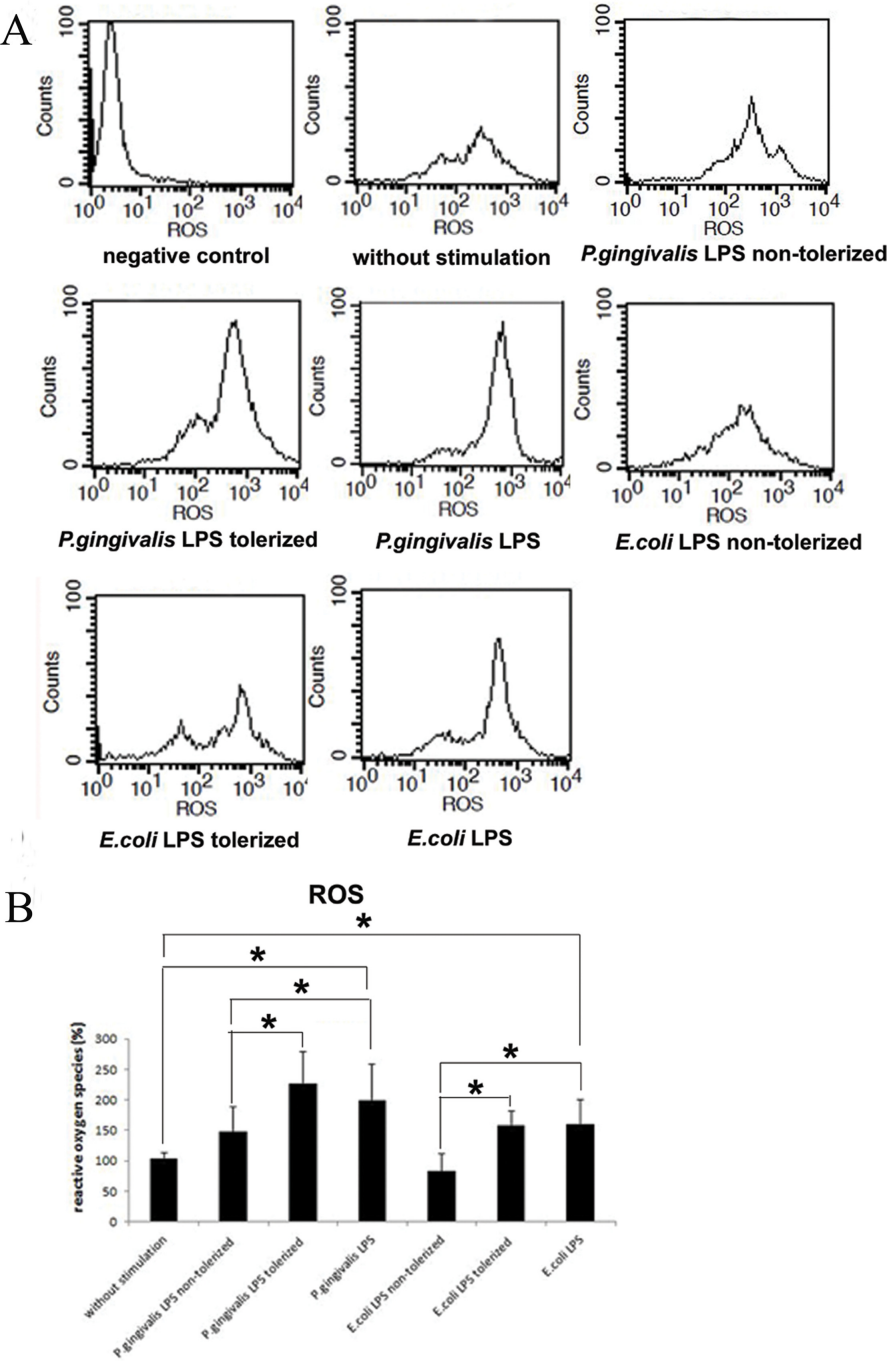
Influences of tolerized THP-1 cells on ROS production in neutrophils. Neutrophils were stimulated with supernatants from 1 μg/ml *P*.*gingivalis* LPS or 1 μg/ml *E*.*coli* LPS tolerized/non-tolerized THP-1 cells for 2 h. *P*.*gingivalis* LPS and *E*.*coli* LPS were served as controls. ROS production was measured by flow cytometry. Data are expressed as mean±SD (n = 5 per group). *p<0.05. One representative result of five independent experiments is shown in (2A).

Using flow cytometry, ROS production from neutrophils was quantified to explore the effect of LPS-tolerized THP-1 cells on neutrophil respiratory burst. 1 μg/ml *P*.*gingivalis* LPS or *E*.*coli* LPS significantly increased ROS production in neutrophils when compared with cells treated with supernatants from THP-1 cells without stimulation (group 1) (p<0.05). However, there were no changes in ROS generation in neutrophils stimulated with cultured medium from LPS-challenged (group 2 and 4) and non-challenged THP-1 cells (group 1) (p>005). Moreover, ROS levels in neutrophils stimulated with supernatants from *P*.*gingivalis* LPS (group 3) or *E*.*coli* LPS-tolerized THP-1 cells(group 5) were significant higher than those stimulated with cultured medium from group 2 or 4, respectively (p>0.05) (Fig 2).

### Decreased Apoptosis of Neutrophils Triggered by Tolerized-THP-1 Cells

Caspase 3, an inactive proenzyme, is a member of the Caspase family, and the active form of the enzyme initiates a protease cascade that causes cell death. In order to evaluate the levels of apoptosis in neutrophils, Caspase 3 activity in neutrophils stimulated with supernatants from THP-1 cells was measured.

**Fig 3:**
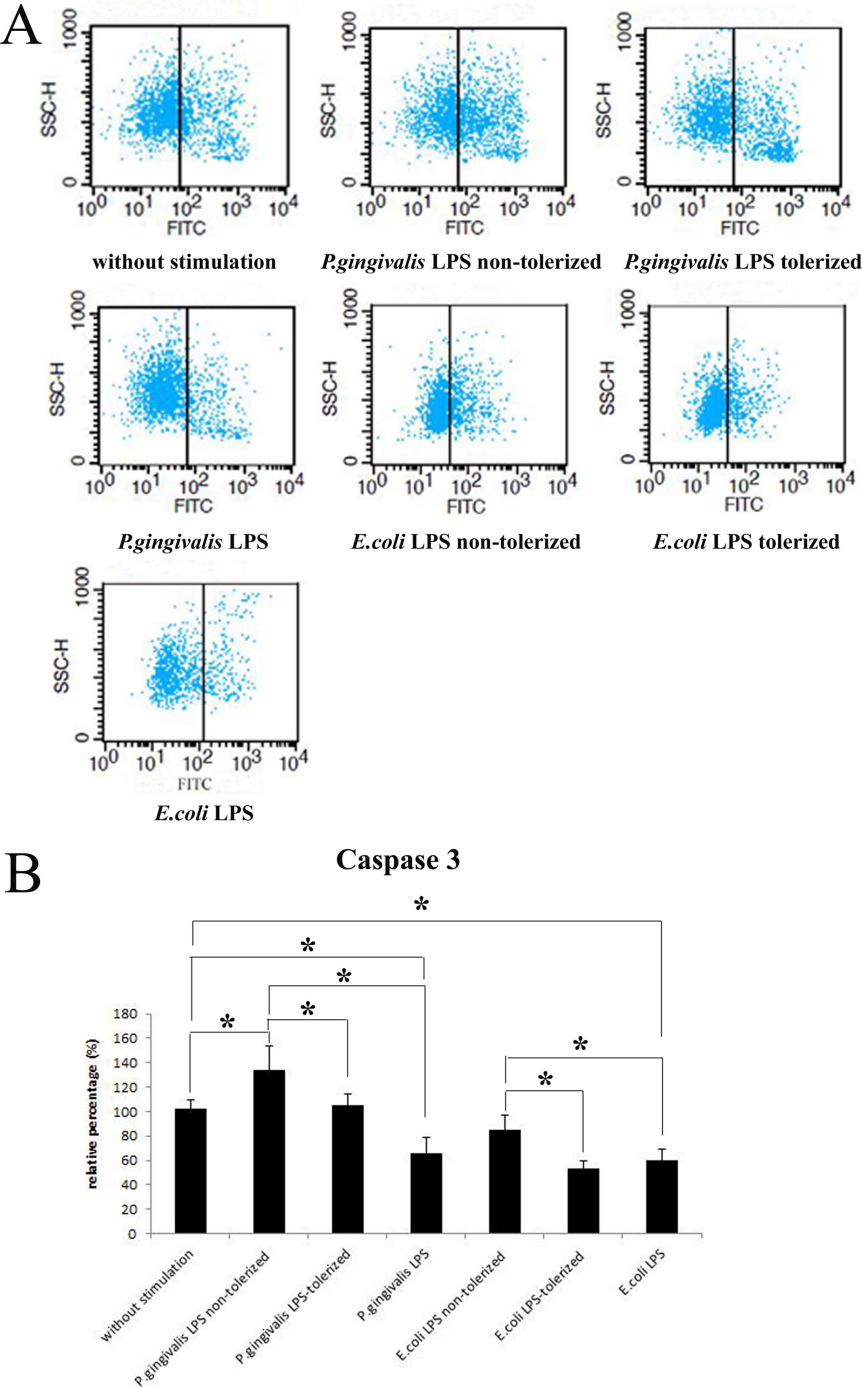
Effects of tolerized THP-1 cells on the expressions of active Caspase 3 in neutrophils. Neutrophils were challenged with supernatants from 1 μg/ml *P*.*gingivalis* LPS or 1 μg/ml *E*.*coli* LPS tolerized/non-tolerized THP-1 cells for 5 h. *P*.*gingivalis* LPS and *E*.*coli* LPS were served as controls. Levels of active Caspase 3 were measured by flow cytometry. Data are expressed as mean±SD (n = 5 per group). *p<0.05. One representative result of five independent experiments is shown in (3A).

Caspase 3 activity in neutrophils cultured with supernatants from 1 μg/ml *P*.*gingivalis* LPS-stimulated THP-1 cells (group 2), were increased significantly in comparison to neutrophils stimulated with medium from unchallenged THP-1 cells (group 1) (p<0.05). Additionally, there were no differences in neutrophils cultured with medium from THP-1 cells stimulated with 1 μg/ml *E*.*coli* LPS (group 4) and fresh medium (group 1) (p>0.05). In addition, there were marked decreases in neutrophils directly challenged with *P*.*gingivalis* LPS or *E*.*coli* LPS (p<0.05). Similar changes could also be noticed between neutrophils treated with cultured medium from THP-1 cells in group 2 and the cells stimulated with *P*.*gingivalis* LPS (p<0.05), as well as *E*.*coli* LPS stimulation (Fig 3A).

After challenge with culture medium from THP-1 cells restimulated with 1 μg/ml *P*.*gingivalis* LPS (group 3) or 1 μg/ml *E*.*coli* LPS (group 5), the level of active Caspase 3 was significantly decreased compared to neutrophils treated with supernatants from THP-1 cells stimulated with *P*.*gingivalis* LPS (group 2) or *E*.*coli* LPS (group 4) only once (p<0.05), which revealed a reduction in neutrophil apoptosis following stimulation with cultured medium from tolerized THP-1 cells (Fig 3A). A representative result of five independent flow cytometry detections is shown in Fig 3B.

### Cytokine Expression Profiles in *P*.*gingivalis* LPS Tolerized-THP-1 Cells

Next, in order to uncover the possible mechanisms responsible for the changes in neutrophil inflammatory responses, a RayBio® Human Cytokine Antibody Array, which allowed for the detection of 170 cytokines, was employed to explore cytokine secretion profiles in *P*.*gingivalis* LPS tolerized- THP-1 cells. Among the total of 170 cytokines, the levels of 7 cytokines were decreased (<2.5-fold) in THP-1 cells upon single *P*.*gingivalis* LPS stimulation, including death receptor 6 (DR6), CD80 and interleukin-1 receptor 2 (IL-1R2), while 38 cytokines expression levels were increased (>2.5-fold), such as chemokine ligand 23 (CCL23), C chemokine ligand 1 (XCL1), intercellular adhesion molecule 1(ICAM 1), ICAM 3, IL-6 and Fas (Table 1).

**Table 1:** Cytokine expression profiles in THP-1 cells stimulated with *P*.*gingivalis* LPS.

single *P*.*gingivalis* LPS stimulation				repeated *P*.*gingivalis* LPS stimulation			
increased cytokine expression (>2.5-fold change)		decreased cytokine expression (>2.5-fold change)		increased cytokine expression (>2.5-fold change)		decreased cytokine expression (>2.5-fold change)	
BLC	3.25	IL-18 BP alpha	2.86	PDGF R alpha	4.81	BLC	2.63
CCL23	2.82	b-NGF	10.07	PDGF R beta	4.07	BMP6	2.78
CNTF	4.06	CCL28	40.13	TGF beta 1	3.03	CCL23	2.70
Eotaxin2	2.69	HPO	5.24	CCL28	9.12	CNTF	3.92
Eotaxin3	2.63	CD80	5.76	b-NGF	4.08	EGF	3.18
FGF-6	3.87	DR6	9.89	CD80	3.85	Eotaxin1	3.12
FGF-7	4.23	IL-1 R2	6.90	DR6	9.03	IFN gamma	2.51
Flt-3 Ligand	4.48			IL-1 R2	8.48	IGFBP1	3.2
IGFBP1	2.79			IL-18 BP alpha	2.75	IL-5	2.54
IGF-1	5.36			PECAM1	6.10	bFGF	2.60
IL-4	4.65			TIE-1	2.60	BTC	2.67
IL-6	2.84					CTACK	4.30
IL-7	3.21					Dtk	4.08
PARC	2.66					Fas	2.53
ANGPT2	2.77					FGF-9	3.18
CTACK	4.83					GITR Ligand	3.20
Dtk	4.07					GITR	3.48
EGFR	4.17					ICAM-1	3.56
ENA-78	4.25					ICAM-3	3.92
Fas	5.07					IGFBP3	2.93
FGF-4	3.04					IL-12 p40	2.72
GITR Ligand	3.98					IL-12 p70	2.52
ICAM-1	2.99					IL-2 R alpha	4.17
ICAM-3	7.39					IL-6 R	2.95
IGFBP3	5.30					I-TAC	3.37
IGF-1 Sr	3.20					XCL1	3.13
IL-1R4	3.94					MIP-3 beta	2.76
IL-1R1	2.70					NT-4	2.55
IL-11	4.26					OPG	2.84
IL-12 p70	2.54					PLGF	3.10
IL-2R alpha	4.34					Sgp130	3.03
I-TAC	5.80					sTNFRII	3.28
XCL1	5.58					sTNFRI	2.89
TECK	5.32					TECK	2.53
IL-21 R	3.37					THPO	2.64
PDGF AA	4.57					TRAIL R3	3.26
PDGF AB	2.54					TRAIL R4	2.60
						VEGF	3.19
						VEGF-D	4.44
						Endoglin	2.96
						ErbB3	2.98
						IL-13Ralpha 2	2.66
						TGF alpha	2.63

**Fig 4:**
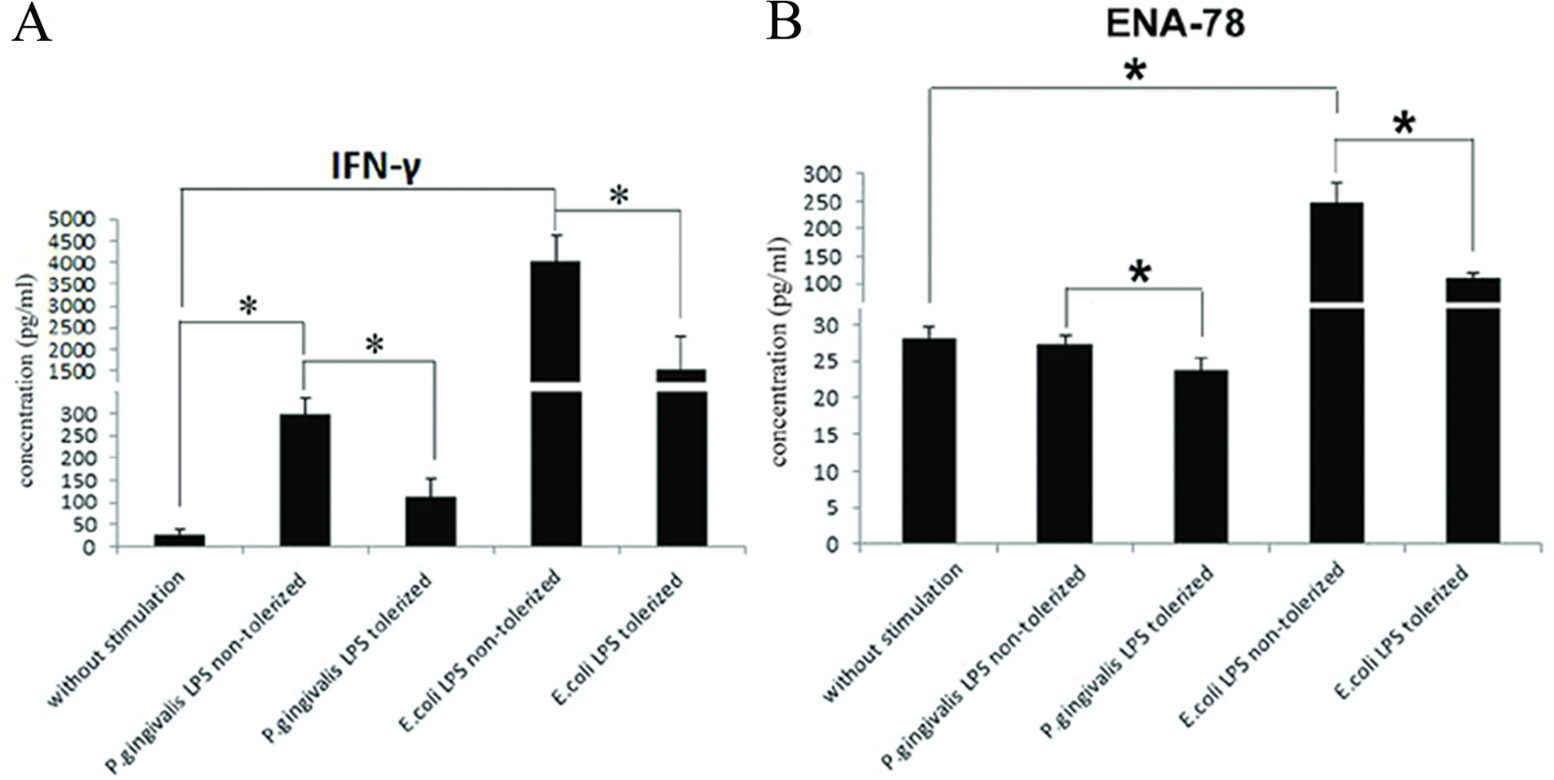
Cytokine production in THP-1 cells stimulated with *P*.*gingivalis* LPS. THP-1 cells were pretreated with medium or 1 μg/ml *P*.*gingivalis* LPS for 24 h, washed, and then incubated with medium or 1 μg/ml *P*.*gingivalis* LPS for another 24 h. Levels of IFN-γ (5A) and ENA-78 (5B) in the cultured supernatants were measured by ELISA. Data are expressed as mean±SD (n = 5 per group). *p<0.05.

THP-1 cells were stimulated with medium or *P*.*gingivalis* LPS as described in the legends to Fig 4. Fold change means that cytokine expression was increased or decreased following treatment with *P*.*gingivalis* LPS.

In comparison to THP-1 cells stimulated with *P*.*gingivalis* LPS only once, 43 cytokines were downregulated (<2.5-fold) in *P*.*gingivalis* LPS-tolerized cells, including Fas, CCL23, IFN-γ, ICAM-1, ICAM-3, XCL1, basic fibroblast growth factor(bFGF), IL-12 p40, IL-12 p70 and osteoprotegerin (OPG). Meanwhile, 11 cytokines were upregulated (>2.5-fold), such as DR6, CD80 and IL-1 R2 (Table 1). Moreover, after *P*.*gingivalis* LPS-restimulation, there were some important cytokines, which did not decrease less than 2.5-fold, but less than 2.0-fold, including ENA-78 (2.22-fold) and fractalkine (2.06-fold). The representative result of three independent experiments is shown in S1 Fig.

### Cytokine Production in *P*.*gingivalis* LPS Tolerized-THP-1 Cells Confirmed by ELISA

In order to verify the cytokine array data, 2 cytokines, IFN-γ and ENA-78, were detected by ELISA.

Stimulation with 1 μg/ml *P*.*gingivalis* LPS or 1 μg/ml *E*. *coli* LPS for 24 h resulted in an enhanced secretion of IFN -γ in THP-1 cells (p<0.05), while a marked increase in production of ENA-78 was observed in cells challenged with *E*.*coli* LPS (p<0.05), but not *P*.*gingivalis* LPS (p>0.05). In addition, the amounts of IFN-γ and ENA-78 induced by *E*.*coli* LPS were much higher than those from the cells treated with *P*.*gingivalis* LPS (p<0.05), which demonstrated a divergence in biochemical and immunobiological properties of *P*. *gingivalis* LPS and *E*.*coli* LPS (Fig 4).

After restimulation with the same LPS for an additional 24 h, the levels of IFN–γ and ENA-78 were decreased markedly in comparison to those from THP-1 cells stimulated with *P*.*gingivalis* LPS or *E*.*coli* LPS only once (p<0.05) (Fig 4).

## Discussion

Neutrophils and monocytes/macrophages are two distinct cell lineages that orchestrate complex functions in innate immunity. Neutrophils kill invading bacteria by releasing ROS and bactericidal granules, which leads to both beneficial infection relief and harmful tissue damage. Multiple signals generated at the site of inflammation, including a wide range of cytokines secreted by monocytes/macrophages, can activate neutrophils [13].

In this study, changes in neutrophil chemotaxis, respiratory burst and apoptosis caused by *P*.*gingivalis* LPS- tolerized THP-1 cells were investigated. Moreover, cytokine expression profiles in tolerized-THP-1 cells, which might be responsible for the observed changes in neutrophils, and altered expression of ENA-78 in endotoxin tolerance were revealed for the first time.

LPS is an important virulence factor of *P*.*gingivalis*, which can cause direct destruction of periodontal tissues and trigger a wide range of immune responses. Protein structure of *P*. *gingivalis* LPS lacks heptose and 2-keto-3-deoxyoctonate, which are unique to classical Gram-negative bacterial LPS, such as *E*.*coli* LPS. Additionally, *P*. *gingivalis* LPS displays unusual amount of lipid A heterogeneity, containing both tetra- and penta-acylated lipid A structures. *P*. *gingivalis* LPS also acts as both a weak agonist and an antagonist aganist Toll-like receptor 4 (TLR4), while *E*. *coli* LPS is a strong agonist of TLR4 and can provoke a strong immune response [14]. Therefore, *E*. *coli* LPS was chosen as a positive control.

Neutrophils are immune effector cells derived from bone marrow. Phagocytosis of neutrophils, cytoplasmic granule liberation, ROS generation and cytokine production contribute to killing bacteria, while also activating monocytes and T cells, which excites a cascade of signal amplification and affects neutrophils simultaneously. Ling observed neutrophils from chronic periodontitis patients constitutively exhibited hyper-reactivity and released more pro-inflammatory cytokines in the presence of *P*.*gingivalis* than those from healthy controls [15].

Once periodontal tissues are invaded by pathogens, neutrophils pass through the endothelial cell lining of vessels and migrate to sites of inflammation [16]. Impaired neutrophil migration hampers host resistance and leads to delayed immune responses. Chemokines, such as IL-8, are key chemical signals that promote neutrophil migration to gingival sulcus. This study indicated the depressed migration of neutrophils upon stimulation with supernatants from tolerized THP-1 cells, which might be caused by cytokines secreted by tolerized THP-1 cells that hamper neutrophil migration. Muthukuru found that the levels of IL-8 decreased in monocytes after restimulation with *P*. *gingivalis* LPS [17]. This tested protein array revealed that chemokine production was suppressed in restimulated cells, including ENA-78, CCL23 and fractalkine.

ENA-78, also known as CXCL5, belongs to a chemokine family and mediates recruitment of neutrophils. Previous studies revealed that ENA-78 secretion in monocytes was increased by *Helicobacter pylori* LPS [18], while degraded recombinant ENA-78 could lead to reduced neutrophil migration [19]. ELISA data confirmed that there was decreased expression of ENA-78 in *P*. *gingivalis* LPS- tolerized THP-1 cells, which might be responsible for suppressed neutrophil migration. CCL23, also termed myeloid progenitor inhibitory factor 1 (MPIF-1) and macrophage inflammatory protein 3 (MIP-3), is originally identified in human aortic endothelial cells and THP-1 cells [20, 21], and displays chemotactic activity on resting T lymphocytes, monocytes and neutrophils via C-C chemokine receptor type 1 (CCR1) [18]. In addition, CCL23 has been reported to suppress neutrophil progenitors and slow turnover in bone marrow [22]. Fractalkine, also named C-X3-C motif chemokine ligand 1(CX3CL1), promotes infiltration of mast cells, neutrophils and macrophages by interacting with CX3C chemokine receptor 1 (CX3CR1) [23], and is a unique chemokine combining properties of chemoattractant and adhesion molecule. Fractalkine and CX3CR1 have been confirmed to act in connective tissue destruction and bone resorption in periapical inflammation [24]. Taken together, these data and previous studies indicated that depressed chemokines secretion in tolerized THP-1 cells might be related to impaired neutrophil migration, which might contribute to restricting inflammation and homeostasis development.

Respiratory burst in neutrophils is an important host defense strategy utilized to kill invading pathogens. At inflammatory sites, NADPH oxidase complex in activated neutrophils is quickly assembled and ROS, including H_2_O_2_, hypochlorite, hydroxyl radicals and singlet oxygen, are generated to eliminate bacteria, eventually damaging surrounding tissues [25, 26]. Both *P*.*gingivalis* and its LPS have been reported to initiate respiratory burst and enhance ROS release in neutrophils [27, 28]. Oxidative levels have also been demonstrated to increase in patients with chronic periodontitis in comparison to healthy individuals [29].

According to the important roles of ROS in inflammation and immune responses, their possible involvement in tolerance was investigated. Levels of ROS were increased in neutrophils after *P*.*gingivalis* LPS stimulations, which was in agreement with Gölz’s study [27]. Interestingly, supernatants from *P*.*gingivalis* LPS stimulated THP-1 cells were able to suppress, but not promote respiratory burst in neutrophils. It was presumed that these changes might be due to some cytokines in the conditioned medium that could inhibit respiratory burst and restrict immune destruction of host tissues. Accumulating evidences indicated that LPS induced IL-27 production in THP-1 cells, and enhanced IL-27 signaling suppressed respiratory burst [30, 31]. Unfortunately, IL-27 was not included in the cytokine chip used in this study, establishing a need for further exploration. Furthermore, ROS levels were higher in neutrophils stimulated with supernatants from tolerized THP-1 cells, which implied the ability of tolerized THP-1 cells to promote respiratory burst and killing bacteria. Conversely, a previous study revealed that ROS depressed pro-inflammatory gene expression depending on the nature of released radical species and the activated signaling pathways [32]. The results from this study suggested that enhanced ROS production in tolerized THP-1 cells might also have an anti-inflammatory effect on regulating rampant immune destruction and serves as an important pathophysiological adaptation upon repeated pathogen stimulation.

Apoptosis has been recognized as a mode of “programmed cell death” and a self-limited preservation method to avoid uncontrolled inflammation and tissue damage triggered by long-lived immune cells. Caspase 3, a marker of apoptosis, is involved in execution pathway of apoptosis and its activation leads to the degradation of chromosome DNA and movement of phosphatidylserine to the outside of the cell membrane [33]. Although not all Caspase family members are expressed in neutrophils, Caspase 3 activity can be detected and synchronized with morphological and biochemical changes during apoptosis [34, 35]. Neutrophil apoptosis is thought to be involved in periodontitis development [36], and Caspase 3 levels in gingival crevicular fluid (GCF) and serum increase in the progression of chronic periodontitis [37].

Numerous studies demonstrated that LPS could suppress neutrophil apoptosis through phosphorylation of AKT, ERK and p38 to upregulate the expression of two antiapoptotic proteins, Mcl-1 and Bcl-2 [38, 39]. In contrary to the effects of LPS, supernatants from THP-1 cells stimulated with *P*.*gingivalis* LPS or *E*.*coli* LPS increased expression levels of Caspase 3, implying the presence of molecules that promote neutrophil apoptosis. In inflammatory responses, some pro-inflammatory cytokines, such as IFN-γ and IL-6, were proved to promote apoptosis and restrict inflammation and immune injury [40–42]. Recent studies reported that IFN-γ promoted cellular apoptosis in vivo and its neutralization prolonged neutrophil survival time in mice [43, 44]. Our present results were consistent with these studies. In addition, IFN-γ could promote apoptosis of monocytes/macrophages, thereby provoking impaired clearance of apoptotic neutrophils and causing persistent inflammation [43].

In addition to proinflammatory cytokines, the protein array analysis revealed decreased expression of DR6 in THP-1 cells stimulated with *P*.*gingivalis* LPS and increased expression in tolerized THP-1 cells. DR6, a member of TNF superfamily, contains extracellular cysteine-rich ligand-binding domains and a cytoplasmic death domain that activates numerous downstream targets, such as caspases, upon receptor oligomerization [44]. Overexpressed DR6 has been shown to induce apoptosis [45]. It should be noted that the regions of DR6 recognized by this cytokine antibody array are extracellular domains in supernatants from THP-1 cells. Soluble extracellular domains without transmembrane regions can’t form signaling complexes and might act as a decoy molecule. Therefore, depressed apoptosis in neutrophils treated with supernatants from tolerized THP-1 cells might be related to the enhanced negative regulation of soluble DR6.

Interestingly, increased expression of Fas, another member of the TNF death receptor superfamily, was disclosed in *P*.*gingivalis* LPS stimulated cells. Exposure to Fas ligand (FasL) causes conformational changes in Fas, which promote the assembly of death-inducing signaling complex (DISC) and activate Caspase 3. Apoptosis is then induced and activated neutrophils and T lymphocytes can be efficiently removed [46]. Similar to DR6, regions of Fas captured by this microarray are also extracellular domains. Soluble form of Fas without transmembrane domains can’t transfer apoptotic signal in cells and conteract apoptosis [47]. In the present study, a decrease of soluble Fas in *P*.*gingivalis* LPS- tolerized THP-1 cells could promote apoptosis, which might be a regulation against decreased production of pro-apoptotic cytokines, such as IFN-γ, and increased expression of anti-apoptotic cytokines, such as soluble DR6.

During inflammation, monocytes/macrophages play an important role in regulating the immune response by secreting a diversity of cytokines, which constitute a complex network to maintain host homeostasis. In this study, the levels of some cytokines, including IFN-γ, ENA-78 and IL-6, were decreased markedly in tolerized THP-1 cells, while production of some others, such as IL-1R2, was increased. Therefore, endotoxin tolerance is not a global decline of all cytokines. Instead, it represents a selective reprogramming of gene and protein expressions [48]. In addition, the effects of endotoxin tolerance are very complicated. Tolerized THP-1 cells not only suppress neutrophil migration and apoptosis, but also promote respiratory burst. Decreased apoptosis and enhanced respiratory burst might contribute to eliminating invading periodontal pathogens, and depressed migration might be related to restricting immune injury. However, long-lived neutrophils could also aggravate tissue destruction. Therefore, homeostasis may be the most important factor in endotoxin tolerance, strongly influencing the severity of inflammation and immune damage.

In summary, *P*.*gingivalis* LPS-tolerized THP-1 cells suppressed neutrophil migration and apoptosis, and contributed to their respiratory burst, which might be related to the changes in cytokine expression patterns in THP-1 cells. Neutrophils and monocytes are not independent actors in immune system and crosstalk between these cells occurs when endotoxin tolerance develops. However, changes of the cytokine network in tolerized cells are complex and not all cytokine varieties were included in this study. Moreover, functions and signaling pathways of cytokines involved in this protein chip were not clear totally and still require further investigation.

## Supporting Information

S1 Fig Cytokine expression profiles in THP-1 cells stimulated with *P*.*gingivalis* LPS were assayed using cytokine arrays. THP-1 cells were pretreated with medium or 1 μg/ml *P*. *gingivalis* LPS for 24 h, washed, and then incubated with medium or 1 μg/ml *P*. *gingivalis* LPS for another 24 h. Cytokine production profiles were explored by RayBio® Human Cytokine Antibody Array G-Series 2000, which included three membranes, Cytokine Array C6, C7 and C8. A representative result of three independent experiments is shown. (A) without stimulation, (B) *P*.*gingivalis* LPS treatment, (C) *P*.*gingivalis* LPS retreatment.
(TIF)
