# Correction: Effects of *Porphyromonas gingivalis* LipopolysaccharideTolerized Monocytes on Inflammatory Responses in Neutrophils

**DOI:** 10.1371/journal.pone.0165568

**Published:** 2016-10-21

**Authors:** The PLOS ONE Staff

There are errors in the Funding section. The correct funding information is as follows: Grant 81370025 (http://www.nsfc.gov.cn/) from National Natural Science Foundation of China and Grant BK20161565 (http://www.jstd.gov.cn/) from Natural Science Foundation of Jiangsu Province was received by YS. Grant 2014–37 (http://www.ec.js.edu.cn/) from Jiangsu Education Department was received by Affiliated Hospital of Stomatology, Nanjing Medical University. The funders had no role in study design, data collection and analysis, decision to publish, or preparation of the manuscript. The publisher apologizes for the errors.

## References

[1] Zhu X-q, Lu W, Chen Y, Cheng X-f, Qiu J-y, Xu Y, et al (2016) Effects of *Porphyromonas gingivalis* LipopolysaccharideTolerized Monocytes on Inflammatory Responses in Neutrophils. PLoS ONE 11(8): e0161482 doi:10.1371/journal.pone.0161482 27536946 10.1371/journal.pone.0161482 PMC4990254

