# Supplementary figures and images for: Effects of Porphyromonas gingivalis LipopolysaccharideTolerized Monocytes on Inflammatory Responses in Neutrophils
Source: PLoS One. 2016 Aug 18;11(8):e0161482. doi: 10.1371/journal.pone.0161482 (PMC4990254; doi:10.1371/journal.pone.0161482)

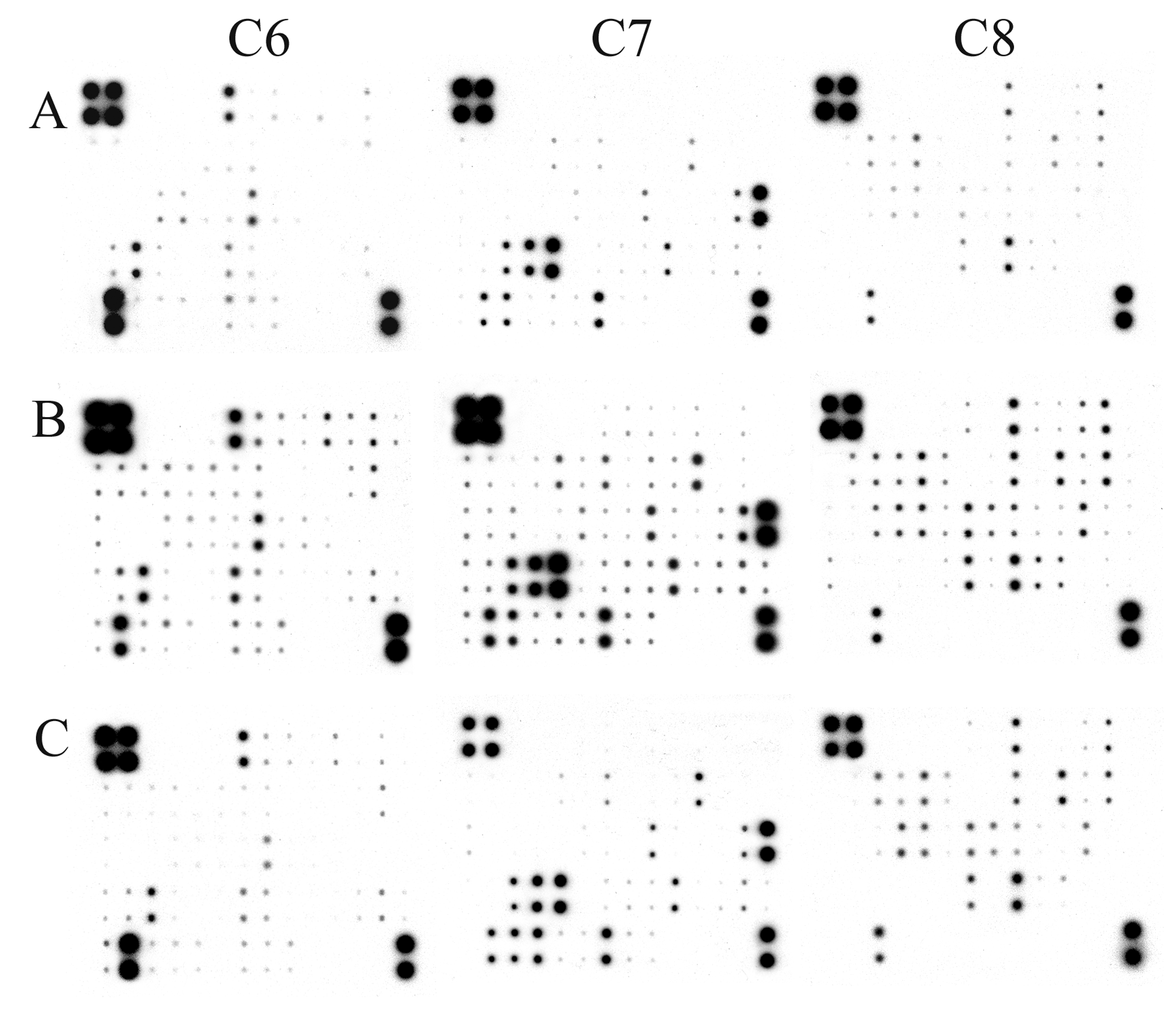

Supplement: S1 Fig — THP-1 cells were pretreated with medium or 1 μg/ml P. gingivalis LPS for 24 h, washed, and then incubated with medium or 1 μg/ml P. gingivalis LPS for another 24 h. Cytokine production profiles were explored by RayBio® Human Cytokine Antibody Array G-Series 2000, which included three membranes, Cytokine Array C6, C7 and C8. A representative result of three independent experiments is shown. (A) without stimulation, (B) P.gingivalis LPS treatment, (C) P.gingivalis LPS retreatment. (TIF) [file pone.0161482.s001.tif]
